# Heat Stress Effect on the Grain Yield of Three Drought-Tolerant Maize Varieties under Varying Growth Conditions

**DOI:** 10.3390/plants10081532

**Published:** 2021-07-27

**Authors:** Uchechukwu Paschal Chukwudi, Funso Raphael Kutu, Sydney Mavengahama

**Affiliations:** 1 Food Security and Safety Niche Area, School of Agricultural Sciences, Faculty of Natural and Agricultural Sciences, North-West University, Mmabatho 2745, South Africa; sydney.mavengahama@nwu.ac.za; 2 Department of Crop Science, Faculty of Agriculture, University of Nigeria, Nsukka 410002, Nigeria; 3 School of Agricultural Sciences, University of Mpumalanga, Mbombela 1200, South Africa; funso.kutu@ump.ac.za

**Keywords:** climate change adaptation, global warming, WEMA maize, Sub-Saharan Africa, *Zea mays* L.

## Abstract

A rise in global temperature will reduce maize yield, particularly in Africa, where maize is a staple food. Therefore, improving maize yield under heat stress will promote food security in the region. The objective of this study was to assess the influence of heat stress on the grain yield of drought-tolerant maize varieties under varied growth conditions. The experimental design used was a 2 × 3 × 3 × 2 factorial fitted into a completely randomized design with four replications. The factors were heat stress, maize variety, soil amendment, and soil type. The results showed a better yield from sandy clay loam over loamy sand soil. Varieties WE5323 and ZM1523 amended with poultry manure gave the best yield under the non-heat and heat-stressed environments, respectively. Heat stress reduced the cob weight, grain weight, grain number, and stover dry weight by 64, 73, 69, and 23%, respectively. Grain number, grain weight, and cob weight were the most informative yield attributes in this study and should be considered in a maize selection program. The ranking for the maize varieties was WE5323 > ZM1523 > WE3128. Drought-tolerant maize varieties can be useful in heat stress mitigation. This information is useful for the simulation of maize yields for heat stress-prone areas in Sub-Saharan Africa.

## 1. Introduction

Increased consumption of animal feed, biofuels, and food will drive a rise in global maize consumption by 23 million tons in 2029, with Sub-Saharan Africa (SSA) consuming over 14 of the 23 million metric tons [1]. Maize is a staple food and the single biggest source of calories in SSA [2].

The expansion of the cultivated land area rather than an increase in yield per area is expected to boost the production of maize in SSA [3,4]. However, this increase might not be sustainable due to various factors, such as climate change and urbanization [1,3]. By 2050, SSA will be at great risk of food insecurity, as her cereal demand is projected to triple, while the current cereal needs are met mostly through importation [5]. Africa is ranked second among the continents to experience a negative impact of climate change [6].

Climate variability and extreme climate events like heat stress remain a threat to food security [7]. Currently, there are indications that the SSA temperature will continue to rise, which will lead to prolonged heat stress on maize [8,9,10] and its subsequent yield losses. Air temperatures across major maize production regions in SSA are expected to rise by over 2.1 °C [11]. In the absence of effective management, each degree Celsius rise in temperature will lead to an approximately 7.4% loss in maize yield [12]. Studies allude to the negative impact of heat stress on maize yield [13,14,15,16].

Yield reduction due to heat stress exacerbates food insecurity and hunger. Satisfying food demands and reducing hunger under extreme climate events like heat stress and a growing population is a global priority. To achieve this goal, there is a need to understand and predict how crops will respond to future climates.

Cairns, Hellin, Sonder, Araus, MacRobert, Thierfelder and Prasanna [11] suggested breeding new maize varieties and adopting new management practices to offset the predicted maize yield decline. Maize breeders have prioritized drought tolerance above heat tolerance [17]. Therefore, evaluating drought-tolerant maize varieties in a heat-stressed environment can facilitate the selection of heat-tolerant lines from the available drought-tolerant varieties. Maize grain yield is a quantitative trait that is expressed differently under varied growth conditions. Hence, the provision of an adequate growth environment is necessary for the actualization of the full genetic potential of an improved maize variety. Maize phenotype is a function of the genotype and the environment for expressing the genotype [18].

Karmakar, et al. [19] associated climate change with land degradation. They argued that a loss of soil carbon will cause poor soil structure, stability, reduced topsoil water retention capacity, and nutrient availability. Pareek [20] postulated that the impact of high temperature on the soil will be region-specific, depending on soil properties and other climatic conditions. Other studies have lent support to the region-specific approach in mitigating the impact of climate change [12,21].

To reduce the negative effects of heat stress on maize yield and ensure food security without environmental degradation, there is a need to re-evaluate the current agronomic practices used for maize production to determine their capability in meeting future needs under extreme climate events. This study adopted a hypothesis that grain yield from drought-tolerant maize varieties exposed to heat stress will differ under varied agronomic practices. The objective of this study was to evaluate the impact of heat stress on grain yield attributes of three drought-tolerant maize varieties grown under different soil types and soil amendments.

## 2. Results

### 2.1. Effects of Heat Stress on the Maize Biological and Economic Yield Attributes

Heat stress had a significant (*p* < 0.05) effect on the maize grain yield attributes studied, except for cob number (NoC) (Table 1). The non-heat stress (NHS) treatment gave higher values than the heat stress (HS) treatment for cob number (NoC), cob length (CL), cob width (CW), cob weight (CWt), grain weight (GWt), shelling percentage (SP), grain number (NoG), 100-seed weight (SWt), and stover dry weight (SDWt). The HS treatment reduced the CL, CW, CWt, GWt, SP, NoG, SWt, and SDWt by 24, 22, 64, 73, 35, 69, 14, and 23%, respectively.

### 2.2. Varietal Performance on the Biological and Economic Yield Attributes of Maize

Maize variety caused significant (*p* < 0.05) variations in all the yield attributes studied, except for the SP (Table 1). Variety ZM1523 gave the highest NoC, which was significantly higher than WE5323. The CL and SWt from ZM1523 were significantly higher than the other two varieties. Variety WE3128 produced the highest CW and SDWt, which were similar to ZM1523 but significantly higher than WE5323. The highest GWt and NoG were obtained from WE5323, which were statistically similar to ZM1523. WE3128 gave the lowest means for CWt, GWt, and NoG.

**Figure 1:**
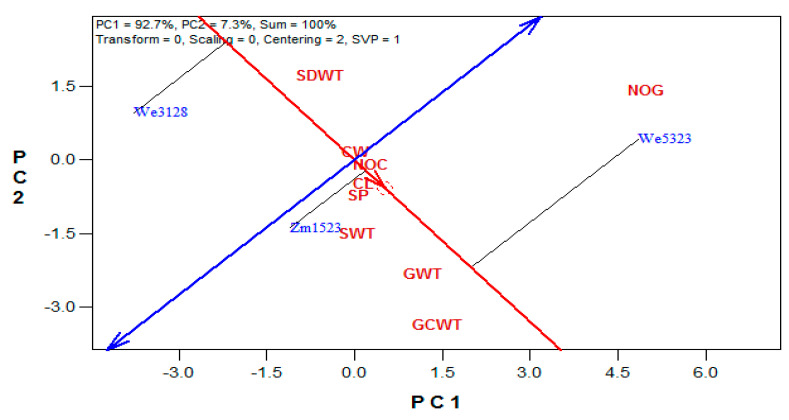
The mean performance vs. stability view of the three maize varieties (NOC = cob number, CL = cob length (cm), CW = cob width (mm), GCWT = cob weight (g plant^−1^), GWT = grain weight (g plant^−1^), NOG = grain number, SP = shelling percentage (%), SWT = 100-seed weight (g plant^−1^), and SDWT = stover dry weight (g plant^−1^)).

**Figure 2:**
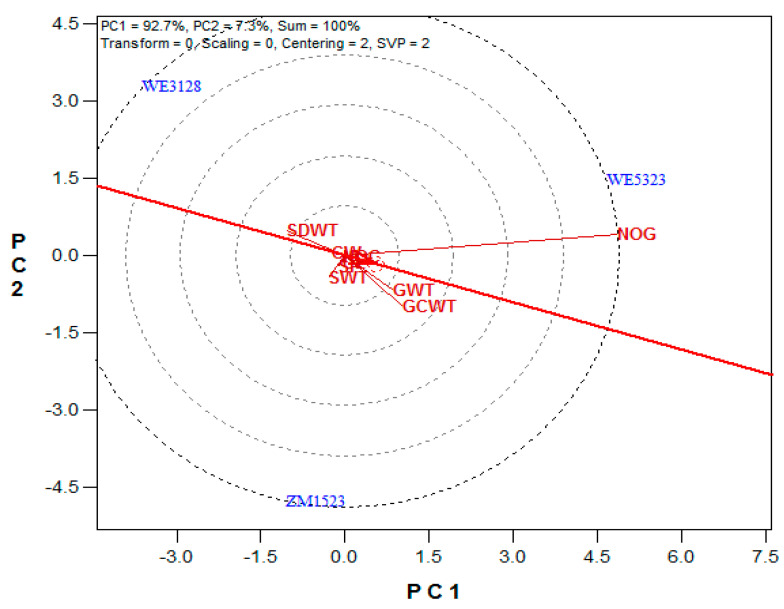
The discriminating and representative abilities of the maize yield attributes on the maize varieties (NOC = cob number, CL = cob length (cm), CW = cob width (mm), GCWT = cob weight (g plant^−1^), GWT = grain weight (g plant^−1^), NOG = grain number, SP = shelling percentage (%), SWT = 100-seed weight (g plant^−1^), and SDWT = stover dry weight (g plant^−1^)).

The maize variety biplot explained 100% (PC1 = 92.7% and PC2 = 7.3%) of the variation among the three maize varieties (Figure 1). The ranking of the maize varieties, based on their mean performance and stability along the average tester axis (ATA, the red line with a single arrow, while the small circle in the red line is the average tester), showed the following order: WE5323 > ZM1523 > WE3128. Variety WE3128 was separated from WE5323 and ZM1523 by the ATA ordinate (the double arrow blue line), which represented the mean of the three maize varieties in all the studied attributes and, thus, separated the maize varieties based on their mean performances. The stability indicator was the black line that connected the maize varieties to the red line. A short black line indicated a more stable performance by a maize variety on the measured attributes. The maize yield attributes studied showed varied discriminating powers and representative abilities, as was indicated by the differences in their vector lengths and angles to the ATA (Figure 2). NoG was the most discriminating attribute, followed by CWt and GWt. The most representative attribute was the GWt, followed by CWt and NoG. The remaining yield attributes were within the first concentric circle from the biplot origin.

### 2.3. Effects of Soil Amendment on the Maize Biological and Economic Yield Attributes

Excluding the SP, soil amendment significantly (*p* < 0.05) influenced the maize yield attributes in this study. The complementary (50:50) application of poultry manure and mineral fertilizer (MPM) produced the highest NoC, CW, and SWt, which were significantly higher than the other soil amendments (Table 1). It was statistically similar to the poultry manure (PM) amendment in the CL, CWt, GWt, NoG, and SDWt. Mineral fertilizer (MF) was significantly lower than the other amendments in the aforementioned yield attributes.

**Figure 3:**
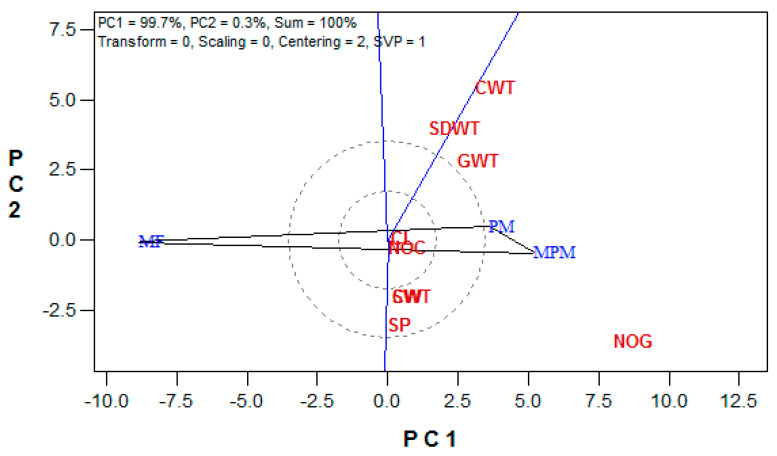
The which-won-where view of the GGE biplot for the soil amendment. (NOC = cob number, CL = cob length (cm), CW = cob width (mm), GCWT = cob weight (g plant^−1^), GWT = grain weight (g plant^−1^), NOG = grain number, SP = shelling percentage (%), SWT = 100-seed weight (g plant^−1^), and SDWT = stover dry weight (g plant^−1^)).

The soil amendment biplot explained 100% (PC1 = 99.7%, PC2 = 0.3%) of the variation in the maize yield attributes (Figure 3). All the studied yield attributes fell within the sector occupied by MPM and PM amendments.

### 2.4. Effects of Soil Type on the Maize Biological and Economic Yield Attributes

Sandy clay loam soil gave a higher NoC, CL, CW, CWt, GWt, SP, NoG, and SDWt than loamy sandy soil, which was higher in SWt. However, sandy clay loam only differed significantly (*p* < 0.05) from loamy sandy soil in CL, CW, NoG, SP, and SDWt (Table 1).

### 2.5. Interaction of Heat Stress, Maize Variety, Soil Amendment, and Soil Type on the Maize Biological and Economic Yield Attributes

The significance levels for the first-, second-, and third-order interactions of the maize yield attributes are presented in Table 2. Nonsignificant differences were observed for SP and NoC for the maize variety × soil amendment interaction and heat stress × soil amendment interaction, respectively. Significant or highly significant differences were observed in the other remaining first-, second-, and third-order interactions.

There were significant interactions of the environment, maize variety, and soil amendment on the studied maize yield attributes. The NHS–ZM1–PM interaction produced the highest NoC, while the NHS–WE5–PM interaction produced the longest CL, which was significantly (*p* < 0.05) higher than most of the other treatment interactions (Table 3). The NoC ranged from 0.8 to 2, while the ranges of 8.3–16.3 cm and 27.1–43.5 mm were obtained for CL and CW, respectively. Under the HS condition, HS–WE3–MPM produced a CW that was statistically similar to the highest CW in the NHS treatment. The NHS–WE5–PM interaction gave the highest CWt and GWt, which were statistically comparable to the NHS–ZM1–MPM, NHS–WE5–MPM, NHS–WE3–MPM, and NHS–ZM1–PM interactions. The HS–ZM1–PM and HS–ZM1–MF interactions produced the highest CWt and GWt under the HS treatment. The CWt ranged from 26.7 g plant^−1^ in HS–WE3–PM to 157.4 g plant^−1^ in NHS–WE5–PM, while the GWt ranged from 15.2 to 121.7 g plant^−1^. The SP ranged from 39.3% in HS–WE3–MPM to 80.0% in the NHS–ZM1–MPM interaction. The highest NoG, 479.2, recorded in the NHS–WE5–MPM interaction, was significantly (*p* < 0.05) higher than the other treatment interactions, except for NHS–WE5–PM, NHS–ZM1–MPM, and NHS–WE3–MPM. The NoG ranged from 64 to 479.2, while the SWt ranged from 14.9 g plant^−1^ in HS–WE5–PM to 31.2 g plant^−1^ in the NHS–ZM1–PM interaction. The SDWt ranged from 59.2 to 134.2 g plant^−1^.

**Figure 4:**
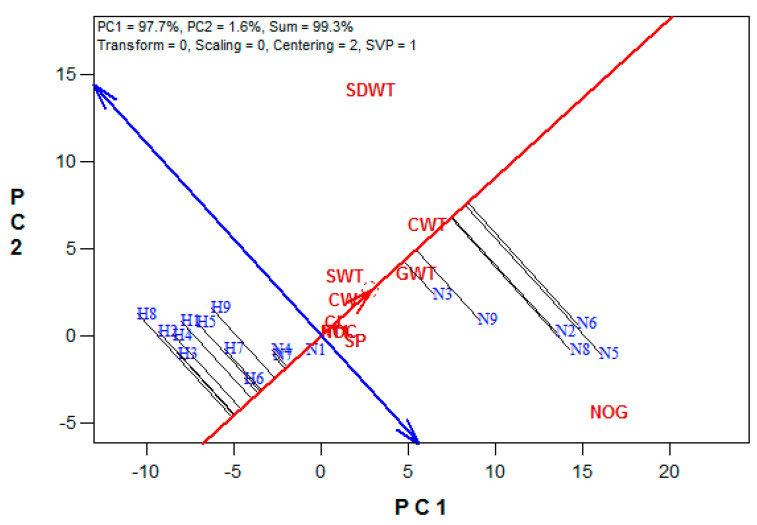
The mean performance vs. stability graph of the treatment interactions. (N1 = NHS–WE3–MF, N2 = NHS–WE3–MPM, N3 = NHS–WE3–PM, N4 = NHS–WE5–MF, N5 = NHS–WE5–MPM, N6 = NHS–WE5–PM, N7 = NHS–ZM1–MF, N8 = NHS–ZM1–MPM, N9 = NHS–ZM1–PM, H1 = HS–WE3–MF, H2 = HS–WE3–MPM, H3 = HS–WE3–PM, H4 = HS–WE5–MF, H5 = HS–WE5–MPM, H6 = HS–WE5–PM, H7 = HS–ZM1–MF, H8 = HS–ZM1–MPM, H9 = HS–ZM1–PM, NOC = cob number, CL = cob length (cm), CW = cob width (mm), GCWT = cob weight (g plant^−1^), GWT = grain weight (g plant^−1^), NOG = grain number, SP = shelling percentage (%), SWT = 100-seed weight (g plant^−1^), and SDWT = stover dry weight (g plant^−1^)).

**Figure 5:**
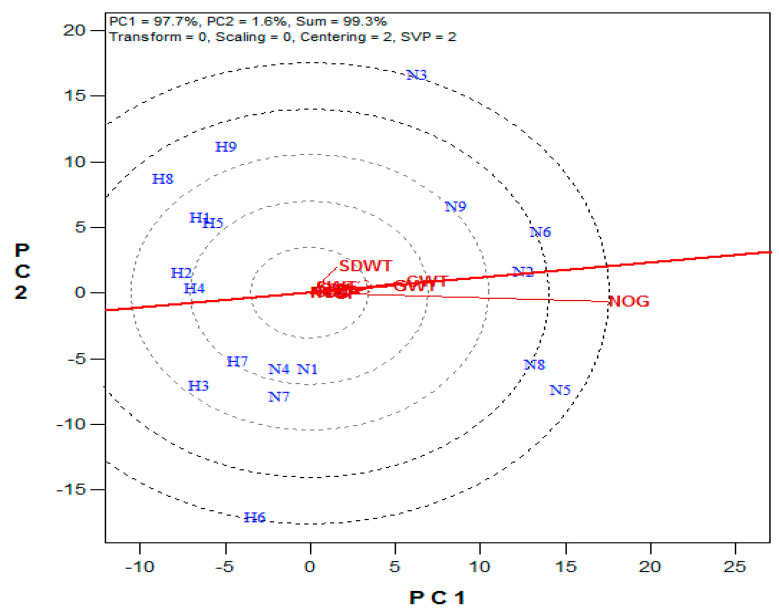
The discriminating and representative abilities of the maize yield attributes. (N1 = NHS–WE3–MF, N2 = NHS–WE3–MPM, N3 = NHS–WE3–PM, N4 = NHS–WE5–MF, N5 = NHS–WE5–MPM, N6 = NHS–WE5–PM, N7 = NHS–ZM1–MF, N8 = NHS–ZM1–MPM, N9 = NHS–ZM1–PM, H1 = HS–WE3–MF, H2 = HS–WE3–MPM, H3 = HS–WE3–PM, H4 = HS–WE5–MF, H5 = HS–WE5–MPM, H6 = HS–WE5–PM, H7 = HS–ZM1–MF, H8 = HS–ZM1–MPM, H9 = HS–ZM1–PM, NOC = cob number, CL = cob length (cm), CW = cob width (mm), GCWT = cob weight (g plant^−1^), GWT = grain weight (g plant^−1^), NOG = grain number, SP = shelling percentage (%), SWT = 100-seed weight (g plant^−1^), and SDWT = stover dry weight (g plant^−1^)).

The mean vs. stability analysis revealed that the first two principal components explained 99.3% (PC1 = 97.7, PC2 = 1.6%) of the variations among the treatment interactions (Figure 4). The ranking of the treatment interactions based on their mean performance and stability across the yield attributes showed that N6, N5, N2, N8, N9, and N3 gave above-average mean performances while N1, N4, N7, H9, H7, H5, H6, H1, H4, H2, H3, and H8 performed below the average mean in the studied maize yield attributes. The most discriminating attribute was NoG, while GWt and CWt were the most representative yield attributes among the studied attributes (Figure 5). The other studied yield attributes centered around the biplot origin.

## 3. Discussion

### 3.1. Effects of Heat Stress on Maize Biological and Economic Yield Attributes

The significant difference in maize yield attributes observed in the two growth environments differing in temperature was attributed to heat stress. The reduced maize yield attributes due to heat stress observed in this study are aligned with the report of Cairns, et al. [22]. Yang, Gu, Ding, Lu and Lu [13] observed reduced dry matter accumulation, grain number, grain weight, and yield in maize due to heat stress. The 23% loss in dry stover weight due to heat stress in this study is close to the 27.9% reported by Yang, Gu, Ding, Lu and Lu [13]. However, their two-year average of 17.9% and 18.7% reported for the grain number and grain weight differed from the 69% and 73% observed in this study. Loss in dry matter accumulation, as well as dry matter partitioning, can lead to reduced grain yield under heat-stressed conditions. Heat stress reduces maize yield through an increased abortion of kernels [16] and premature cessation of grain filling [23]. These disruptions result in lower grain numbers and weight. Pollen sterility and kernel abortion due to heat stress reduce the grain number (sink strength) for assimilate partitioning in maize. The denaturing of important enzymes involved in photosynthesis [24] and disruption of other major physiological processes [25] can lead to a reduced grain-filling period in maize. A lesser amount of assimilates are partitioned to the economic parts under a reduced grain-filling period. Heat stress-induced dry matter loss in maize often results in grain yield loss [13,15,26].

**Figure 6:**
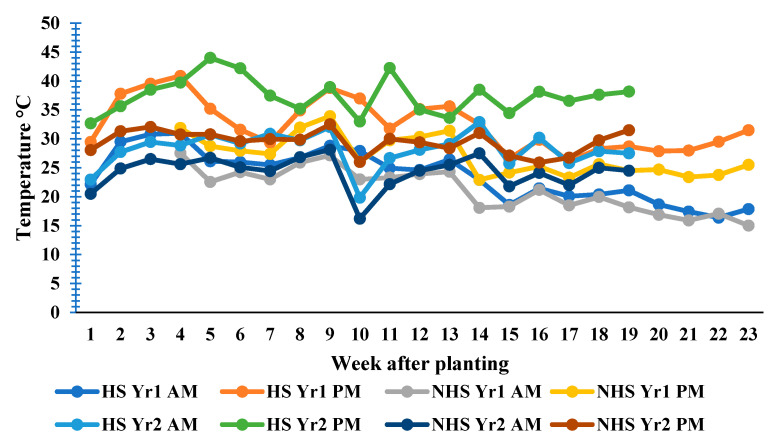
Weekly temperature records for HS and NHS environments for the two seasons plantings (Yr1 = 2018/2019, Yr2 = 2019/2020, AM = morning, and PM = afternoon).

Higher yield loss occurs at temperatures above 35 °C [14]. In this study, temperatures during the reproductive phase ranged from 32 to 37 °C in Season 1 and 34 to 39 °C in Season 2 for the HS treatment, while the NHS treatment had 23–31 °C in Season 1 and 27–31 °C in Season 2 (Figure 6). The recorded high temperatures in the HS environment contributed to the observed yield loss in this study. Cultivation of a genotype in a less-favorable environment leads to yield loss. Edaphic, as well as environmental factors, often delineate the niche of most plant species [26].

### 3.2. Varietal Performance on the Biological and Economic Yield Attributes of Maize

GGE biplot had been used to explain yield variations in many economic crops, like *Triticum aestivum* L. [27], *Saccharum officinirum* L. [28], *Zea mays* L. [29], and *Telfaria occidentalis* L. [30]. GGE biplot is an efficient tool for interpreting genotype by trait interactions [31], as well as predicting the mean genotype yield per specific environments [29]. In this study, the GGE biplot was used to identify the attributes that explained more variations in the studied treatments (discriminating vs. representative abilities) and the treatments that produced the best attributes (the mean performance vs. stability). The mean vs. stability test identified variety WE5323 as the best variety, followed by ZM1523, based on the studied yield attributes. These two varieties gave means that were above the population mean (before the blue line in Figure 1), while a below the population mean performance was obtained from variety WE3128. The high instability (the longest black line in Figure 1) observed in WE5323 may be due to its high NoG.

Though statistically similar in most yield attributes, the high NoG obtained from WE5323 may have influenced its ranking ahead of ZM1523 in this study, because NoG was the most discriminating attribute among the studied yield attributes. The NoG produced the longest vector (line from the biplot origin), thus explaining more variations among the three maize varieties than any other attribute in this study. The location of some of the yield attributes close to the biplot origin is a sign that the three drought-tolerant maize varieties performed evenly on them. Yan, et al. [32] reported that an attribute that is close to the biplot origin should not be used in the selection, as it provides little or no information on the variation within the studied population. The identification of NoG, GWt, and CWt as the leading yield attributes for maize selection in this study agreed with earlier reports [22,33] on maize.

The genetic variations inherent in the studied varieties were expressed in their yield attributes. Genetic variation within a germplasm is important for preserving biodiversity. Variations within a maize population, as observed in this study, was also found in previous studies on maize [18,34,35,36,37]. Though these varieties were initially bred for drought and disease tolerance [38], they may be useful in heat stress mitigation. The development of a germplasm adapted to heat stress-prone regions has been a key strategy for reducing yield losses in crops [39].

### 3.3. Effects of Soil Amendment on Maize Biological and Economic Yield Attributes

Mineral fertilizers release nutrients fast and are less bulky to handle compared to organic manures. However, soil degradation, nutrient leaching and environmental pollution are some ills associated with mineral fertilizers. A balanced nutrients supply, improved soil structure, and water retention capacity are some benefits linked to organic manure usage. These benefits promote the use of organic manure as a soil amendment [40].

Martínez, et al. [41] opined that, to protect the environment and improve the soil quality, organic manure-based amendments can replace mineral fertilizers without a yield loss penalty. Geng, et al. [42] reported that the replacement of 25% of the total nitrogen of mineral fertilizers with organic manure produced the best maize yield increase. Zhang, et al. [43] considered the substitution of 30% of the total nitrogen fertilizer with compost as an effective way of reducing nitrogen loss and improving soil fertility, as well as achieving a high yield in maize. These reports corroborated the findings in this study, where MPM produced the best maize yield.

In this study, the which-won-where graph ranked the soil amendments as follows: MPM ≥ PM > MF. The positioning of MF in the sector with no attributes and directly opposite the MPM, which won in all the attributes, implied a low performance from the MF amendment. Yan [44] stated that entries occupying the polygon vertex in each sector are the best-performing entries for the testers within the sector. The double vertices observed in the sector that contained MPM and PM amendments indicated similarities in the good performances from the two amendments. However, the lengthy vector from the MPM amendment signifies a higher mean performance over the PM amendment.

Geng, Cao, Wang and Wang [42] recommended that appropriate organic substitution is beneficial to the yield but cautioned that excessive organic fertilizer substitution may lead to insufficient nitrogen and the accumulation of phosphorus and potassium. Therefore, the integrated use of mineral fertilizers with organic manure is a sustainable approach for efficient nutrient usage. This enhances the efficiency of the mineral fertilizers while reducing the nutrient losses [45,46].

### 3.4. Effects of Soil Type on Maize Biological and Economic Yield Attributes

The differences in the two soil properties may have influenced the variations in the maize yield attributes obtained in them (Table A1). Fang and Su [47] observed a higher nitrogen uptake and maize yield in sandy loam soil compared with loamy sand and sandy soils. They attributed the higher performance from sandy loam soil to higher organic matter, nutrient concentration, and silt/clay contents. In this study, the sandy clay loam soil had higher silt/clay content; organic carbon; total nitrogen; and extractable Ca, Mg, K, and Na compared to the loamy sand soil. These properties may have influenced the better sandy clay loam performance in maize yield. Moral and Rebollo [48] suggested that these properties may have a high influence on soil fertility.

Ahmadi, et al. [49] suggested that high clay contents in the soil promote transpiration and crop evapotranspiration, which leads to a higher nitrogen uptake and yield. Adeyemo, Akingbola and Ojeniyi [37] also reported a higher maize yield in soil containing a high clay content. These reports corroborated the findings in this study where the soil properties influenced the maize grain yield. Rao, et al. [50] attributed the low productivity in sandy soil to a low organic matter content, nutrient preservation, and water retention capacities.

### 3.5. Interaction of Heat Stress, Maize Variety, Soil Amendment, and Soil Type on Maize Biological and Economic Yield Attributes

Crop yield is influenced by its genetic component and management practices [26,51]. Crop management ranges from choice of site vis-à-vis soil properties, as well as environmental factors and nutrient supply options to time of harvest. To maximize maize production, Belay and Adare [35] recommended the adoption of appropriate agronomic practices, including the use of improved varieties. Improved crop growth and yield require an adequate supply of nutrients [52] and the absence of stress-limiting factors like heat stress. Heat stress is one of the major factors that limit agricultural productivity, including maize yield [13,14,15,16].

In this study, the NHS environment provided favorable conditions for maize growth and development, leading to a better performance in the studied yield attributes. On the other hand, the HS environment placed limitations on the actualization of maize yield potential, as the plants adjusted to tolerate the stress. The contribution of stress tolerance to yield improvement is the difference between yield potential and actual yield [53]. The three drought-tolerant maize varieties assessed in this study showed varied grain yields under the NHS and HS conditions. The identification of high-yielding maize varieties under the HS environment is necessary to ensure food security, as many studies have predicted an increased occurrence of heat stress due to global warming [8,9,54,55].

The ranking of the treatment interactions showed an overall better ranking from the NHS plants compared to the HS plants. Variety WE5323 grown under the PM amendment gave the best performance. NHS–WE5–PM (N6) produced the highest CL, CW, CWt, and GWt. Its mean for NoG, SP, and SWt were similar to the highest obtained means in these attributes. It was followed by NHS–WE5–MPM (N5), which produced the highest NoG. The best above-average mean performance for varieties WE3128 and ZM1523 was under the MPM amendment. The three maize varieties produced their lowest grain yields with the MF amendment in the NHS environment. In the HS environment, variety ZM1523 amended with PM, ranked first. The highest rankings for varieties WE5323 and WE3128 were under MPM and MF amendments, respectively. These variations in the maize varieties under different growth conditions in this study were aligned with other studies [34,35,36,37] that reported significant variations in maize yield attributes due to the interactions of climate and agronomic practices.

The top two ranked treatment interactions (H9 and H7) in the HS environment produced the highest CL, CWt, and GWt. The highest NoG, as well as the lowest SWt produced in the HS environment, were obtained from H6. Similarly, the lowest NoG and the highest SWt produced in the HS environment came from H8. This association between NoG and SWt suggests a compensatory seed weight gain in maize under the HS condition. A similar compensatory seed weight gain was reported in maize under drought stress and salt stress [51,56]. A probable explanation for this compensatory seed weight gain may be that all the assimilates partitioned to the sink may have been used to fill the few set seeds, resulting in a higher SWt.

## 4. Materials and Methods

### 4.1. Description of the Study Site

The experiments were performed within the North West Province of South Africa, which is classified as a semi-arid region with an average daily minimum and maximum temperatures of 0.9 and 32 °C, respectively, with approximately 300–600 mm of rainfall annually [55]. Repeated greenhouse experiments were performed in the 2018/2019 and 2019/2020 summer planting seasons at the North-West University Experimental Farm (−25.7902166, 25.6187922 3.5 mi). Soil (0–20 cm depths) used for the pot experiments were from uncultivated fields at Molelwane (−25.7902166, 25.6187922 3.5 mi) and Tlapeng (−25.7297143, 25.4324561 13 mi), classified as Hutton and Arcadia forms, respectively [56]. The two soil properties are presented in Table A1. Mineral fertilizers and poultry manure from the layers were provided by the North-West University Agricultural Farm, Mafikeng campus, while the maize varieties were sourced from the Agricultural Research Council, Grain-Crops Potchefstroom Station. All the locations are within the North West Province, South Africa.

### 4.2. Treatments, Experimental Design, and Cultural Practices

A factorial in a completely randomized design with 4 replications was used for this study. Four factors were combined in all possible combinations to generate 36 (2 × 3 × 3 × 2) treatment interactions. Factor A was the heat stress (HS) and non-heat stress (NHS) conditions. The HS effect was achieved by using two growth structures differing in temperature. A transparent plastic greenhouse was used to create heat stress (HS) conditions, while a net shade house was used to achieve non-heat stress (NHS) conditions. The temperature within the plastic greenhouse could rise and drop with the temperature outside the greenhouse. The morning and mid-day temperatures in both growth structures were recorded throughout the period of the study (Figure 6).

Factor B was drought-tolerant maize varieties involving three-way Water-Efficient Maize for Africa hybrids: WE3128, WE5323, and an open-pollinated variety ZM1523. The three maize varieties were medium-maturing varieties with a range of 117–120 days. Factor C represented soil amendment, comprising the application of sole poultry manure (PM), mineral fertilizers (MF), and a complementary (50:50) application of poultry manure and mineral fertilizer (MPM). The mineral fertilizers included NPK (13:7:10 [30] + 0.5% Zn + 5% S + 3% Ca) and lime ammonium nitrate (28% N), while the poultry manure came from mature layer birds. Each of the soil amendments supplied 180 kg N ha^−1^. Factor D comprised two soil types: loamy sand (LS) soil (Hutton form) from Molelwane and sandy clay loam (SCL) soil (Arcadia form) from Tlapeng.

Surface soils (0–20 cm depths) were excavated, air-dried, sieved, homogenized, and 12 kg were weighed into each plastic planting pot (30 cm top × 28 cm height × 21 cm base), perforated at the base for drainage. The 12-kg weighed soil and the organic amendments were thoroughly and separately mixed on a polyethene sheet and, thereafter, transferred into each pot based on the treatment. The pots were well-irrigated to approximately 80% field capacity and allowed to stand for two weeks for mineralization of the poultry manure before the sowing of the maize seeds. Three maize seeds were sown per pot, but the seedlings thinned to one per pot at 2 weeks after planting (WAP). The mineral fertilizer rates of 180 and 90 kg N ha^−1^ for MF and MPM, respectively, were applied in a split dosage, with 50% applied first at planting using NPK fertilizer and the remaining 50% supplied using lime ammonium nitrate (28% N) at 7 WAP. Weeds were manually removed and the pots well-irrigated.

### 4.3. Measurement of Plant Response Attributes

After harvest, the cob number per plant (NoC) was recorded. The cobs were dried under shade to achieve a 12% moisture content, while the shoots were oven-dried at 65 °C to a constant weight for determination of the stover dry weight in grams (SDWt). Cob length (CL) and cob width (CW) were measured in cm and mm using a measuring tape and digital Vernier calliper (153-006-11, ACCUD Austria), respectively. Each cob was weighed to determine the cob weight in gram (CWt). Afterwards, the cobs were shelled, and the grain weight (GWt) was determined. Shelling percentage (SP) was calculated as:

(1) $$\mathrm{Shelling}\;\mathrm{percentage}\;\left(\%\right)=\frac{\mathrm{Grain}\;\mathrm{weight}\;\left(g\right)}{\mathrm{Cob}\;\mathrm{weight}\;\left(g\right)}\;\times \;100$$

The grains were counted (NoG) using the Numigral seed counter, and 100 seeds from each treatment interaction were weighed to get the 100-seed weight (SWt).

### 4.4. Data Analysis

An analysis of variance (ANOVA) test was performed on the collected data following the procedure for factorial in a completely randomized design using GenStat Software (VSN Int. Ltd., Hemel Hempstead, UK). Protected Fisher’s least significant difference was used as the post hoc test for mean separation at *p* ≤ 0.05. GGE biplot software [31] was used to plot the which-won-where graph, the mean performance vs. stability graph, and discriminating vs. representative abilities biplot of the maize yield attributes. The differences in the maize yield attributes due to the heat stress effect were calculated as:

(2) $$\mathrm{Difference}\;\left(\%\right)=\frac{\mathrm{Ta}\;-\;\mathrm{Tb}}{\mathrm{Tb}}\;\times \;100$$

where Ta is the mean of attribute X in the non-heat stress environment, and Tb is the mean of the same attribute in the heat stress environment.

## 5. Conclusions

In this study, yield variations occurred within and between varieties when grown under varied conditions. The ranking of the treatment interactions showed an overall better ranking from the non-heat-stressed plants compared to the heat-stressed plants. Heat stress had a depressive impact on the maize yield attributes, reducing the cob length, cob width, cob weight, grain weight, shelling percentage, grain number, 100-seed weight, and stover dry weight by 24, 22, 64, 73, 35, 69, 14, and 23%, respectively. Heat stress promoted a compensatory seed weight gain in maize. The sandy clay loam soil improved the maize yield attributes more than the loamy sandy soil, while the ranking for soil amendments in improving the maize yield attributes was MPM ≥ PM > MF. The ranking of the maize varieties based on the mean performance on the studied yield attributes was WE5323 > ZM1523 > WE3128. Under the non-heat stress environment, variety WE5323 got its best ranking with the PM amendment, while ZM1523 and WE3128 recorded their highest performances with the MPM amendment. However, in the heat-stressed environment, variety ZM1523 amended with PM ranked first, while the highest rankings for varieties WE5323 and WE3128 were under the MPM and MF amendments, respectively. In the ranking of the treatment interactions, grain number was the most discriminating attribute, while grain weight and cob weight were the most representative attributes. Drought-tolerant maize varieties can be useful in heat stress mitigation. Hence, consideration should be given to the interaction of variety, soil amendment, and soil type in maize yield improvement under heat stress condition, as maize responds differently to changes in these factors. This information is useful for the simulation of maize yield for heat stress-prone areas in Sub-Saharan Africa.

## Figures and Tables

**Table 1:** Main effects of heat stress, maize variety, soil amendment, and soil type on the maize yield attributes.

	NoC	CL	CW	CWt	GWt	SP	NoG	SWt	SDWt
		cm	mm	g Plant^−1^	g Plant^−1^	%		g Plant^−1^	g Plant^−1^
**Heat stress**									
NHS	1.3 ^a^	13.5 ^a^	39.7 ^a^	112.3 ^a^	86.7 ^a^	75.5 ^a^	339.0 ^a^	25.6 ^a^	94.7 ^a^
HS	1.3 ^a^	10.3 ^b^	31.0 ^b^	40.8 ^b^	23.8 ^b^	48.8 ^b^	105.4 ^b^	22.1 ^b^	73.2 ^b^
F-LSD _(0.05)_	0.2	0.7	1.4	7.4	6.6	5.0	30.5	1.4	7.1
**Maize variety**									
WE3128	1.3 ^b^	11.5 ^b^	36.3 ^a^	69.5 ^b^	49.9 ^b^	61.9 ^a^	205.9 ^b^	23.5 ^b^	89.4 ^a^
WE5323	1.2 ^b^	11.6 ^b^	34.3 ^b^	79.9 ^a^	58.3 ^a^	61.2 ^a^	245.4 ^a^	21.8 ^c^	79.8 ^b^
ZM1523	1.5 ^a^	12.6 ^a^	35.4 ^ab^	80.1 ^a^	57.6 ^ab^	63.3 ^a^	215.1 ^ab^	26.2 ^a^	82.7 ^ab^
F-LSD _(0.05)_	0.2	0.9	1.8	9.0	8.1	6.1	37.4	1.7	8.7
**Soil amendment**									
MF	1.2 ^b^	10.9 ^b^	34.0 ^b^	48.8 ^b^	32.9 ^b^	62.5 ^a^	151.2 ^b^	22.8 ^b^	71.2 ^b^
MPM	1.5 ^a^	12.5 ^a^	37.0 ^a^	90.4 ^a^	67.2 ^a^	63.3 ^a^	265.7 ^a^	25.4 ^a^	89.7 ^a^
PM	1.3 ^b^	12.4 ^a^	34.9 ^b^	90.3 ^a^	65.7 ^a^	60.6 ^a^	249.5 ^a^	23.4 ^b^	91.0 ^a^
F-LSD _(0.05)_	0.2	0.9	1.8	9.0	8.1	6.1	37.4	1.7	8.7
**Soil type**									
Loamy Sand	1.3 ^a^	11.6 ^b^	34.3 ^b^	73.4 ^a^	52.8 ^a^	59.6 ^b^	206.8 ^b^	24.1 ^a^	79.5 ^b^
Sandy Clay Loam	1.3 ^a^	12.3 ^a^	36.4 ^a^	79.7 ^a^	57.8 ^a^	64.6 ^a^	237.5 ^a^	23.6 ^a^	88.4 ^a^
F-LSD _(0.05)_	0.2	0.7	1.4	7.4	6.6	5.0	30.5	1.4	7.1

Means with the same letter(s) within the same column are not significantly different at *p* ≤ 0.05, NHS = non-heat stressed, HS = heat stressed, MF = Mineral fertilizer, MPM = mineral fertilizer with poultry manure, PM = poultry manure, NoC = cob number, CL = cob length (cm), CW = cob width (mm), CWt = cob weight (g plant^−1^), GWt = grain weight (g plant^−1^), NoG = grain number, SP = shelling percentage (%), SWt = 100-seed weight (g plant^−1^), and SDWt = stover dry weight (g plant^−1^).

**Table 2:** First-, second-, and third-order interactions of heat stress, maize variety, soil amendment, and soil type on the maize yield attributes.

Treatment Interactions	NoC	CL	CW	CWt	GWt	NoG	SP	SWt	SDWt
HS.MV	**	**	**	**	**	**	**	**	*
HS.SA	**	**	**	**	**	**	**	**	**
MV.SA	**	**	**	*	*	*	n.s.	**	*
HS.ST	n.s.	**	**	**	*	*	**	*	**
MV.ST	*	**	**	**	*	**	*	*	*
SA.ST	**	**	**	**	**	**	**	**	**
HS.MV.SA	**	**	**	**	**	**	*	**	**
HS.MV.ST	*	**	**	**	**	**	**	**	**
HS.SA.ST	*	**	**	*	**	*	**	**	*
MV.SA.ST	**	**	**	**	**	**	**	*	**
HS.MV.SA.ST	*	**	**	**	**	**	**	**	**

n.s. = not significant, * = significant at 5% probability level, ** = significant at 1% probability level, HS = heat stress, MV = maize variety, SA = soil amendment, ST = soil type, NoC = cob number, CL = cob length (cm), CW = cob width (mm), CWt = cob weight (g plant^−1^), GWt = grain weight (g plant^−1^), NoG = grain number, SP = shelling percentage (%), SWt = 100-seed weight (g plant^−1^), and SDWt = stover dry weight (g plant^−1^).

**Table 3:** Interaction of heat stress, maize variety, and soil amendment on the maize yield attributes.

SN	Treatment Interactions	NoC	CL	CW	CWt	GWt	NoG	SP	SWt	SDWt
			cm	mm	g Plant^−1^	g Plant^−1^		%	g Plant^−1^	g Plant^−1^
N1	NHS–WE3–MF	1.0 ^cd^	11.7 ^d–g^	36.0 ^c–f^	58.0 ^cd^	43.1 ^cd^	215.6 ^de^	71.8 ^a–c^	20.8 ^f–i^	77.2 ^d–g^
N2	NHS–WE3–MPM	1.4 ^bc^	15.9 ^a^	39.8 ^a–c^	141.9 ^a^	113.4 ^a^	430.9 ^ab^	79.8 ^a^	26.6 ^a–e^	108.9 ^b^
N3	NHS–WE3–PM	1.5 ^a–c^	13.0 ^b–e^	42.4 ^ab^	114.6 ^b^	87.1 ^b^	315.3 ^c^	71.5 ^a–c^	30.1 ^ab^	134.2 ^a^
N4	NHS–WE5–MF	1.0 ^cd^	9.5 ^gh^	38.3 ^a–d^	54.2 ^c–e^	38.6 ^c–e^	183.3 ^d–f^	71.2 ^a–c^	21.3 ^f–i^	69.4 ^fg^
N5	NHS–WE5–MPM	1.5 ^a–c^	15.2 ^ab^	41.8 ^ab^	143.1 ^a^	110.7 ^a^	479.2 ^a^	77.3 ^a^	23.2 ^d–g^	98.4 ^b–d^
N6	NHS–WE5–PM	1.1 ^b–d^	16.3 ^a^	43.5 ^a^	157.4 ^a^	121.7 ^a^	445.6 ^ab^	77.3 ^a^	27.8 ^a–d^	112.4 ^ab^
N7	NHS–ZM1–MF	1.0 ^cd^	10.4 ^e–h^	35.5 ^c–f^	51.2 ^c–f^	36.7 ^c–f^	186.0 ^d–f^	72.7 ^ab^	20.5 ^g–i^	66.7 ^fg^
N8	NHS–ZM1–MPM	1.6 ^ab^	14.8 ^a–c^	40.5 ^a–c^	151.4 ^a^	121.3 ^a^	442.5 ^ab^	80.0 ^a^	28.8 ^a–c^	85.1 ^c–f^
N9	NHS–ZM1–PM	2.0 ^a^	15.0 ^ab^	39.6 ^a–c^	138.4 ^ab^	108.0 ^a^	352.2 ^bc^	77.6 ^a^	31.2 ^a^	100.5 ^bc^
H1	HS–WE3–MF	1.2 ^b–d^	11.3 ^e–g^	30.9 ^f–h^	43.4 ^d–g^	24.6 ^d–g^	99.0 ^fg^	49.6 ^de^	27.6 ^a–e^	79.8 ^c–g^
H2	HS–WE3–MPM	2.0 ^a^	8.3 ^h^	36.0 ^c–f^	32.7 ^fg^	16.1 ^fg^	81.7 ^g^	39.3 ^e^	18.8 ^h–j^	76.7 ^d–g^
H3	HS–WE3–PM	1.0 ^cd^	9.2 ^gh^	30.8 ^f–h^	26.6 ^g^	15.2 ^g^	105.4 ^fg^	58.0 ^b–d^	16.9 ^ij^	60.0 ^g^
H4	HS–WE5–MF	1.5 ^a–c^	10.1 ^f–h^	31.4 ^f–h^	35.5 ^d–g^	20.3 ^e–g^	91.0 ^fg^	52.6 ^de^	22.8 ^e–h^	71.8 ^e–g^
H5	HS–WE5–MPM	1.3 ^b–d^	10.9 ^e–g^	33.2 ^d–g^	46.2 ^c–g^	27.6 ^d–g^	108.2 ^fg^	53.5 ^de^	26.8 ^a–e^	82.3 ^c–f^
H6	HS–WE5–PM	0.8 ^d^	12.2 ^d–f^	28.4 ^gh^	68.9 ^c^	49.2 ^c^	264.6 ^cd^	56.4 ^cd^	14.9 ^j^	68.9 ^fg^
H7	HS–ZM1–MF	1.2 ^b–d^	12.4 ^c–f^	32.6 ^e–g^	50.6 ^c–g^	34.1 ^c–g^	136.7 ^e–g^	59.2 ^b–d^	24.0 ^c–g^	59.2 ^g^
H8	HS–ZM1–MPM	1.6 ^ab^	10.1 ^f–h^	27.1 ^h^	28.4 ^fg^	15.6 ^g^	64.0 ^g^	44.3 ^de^	25.6 ^b–f^	91.4 ^b–e^
H9	HS–ZM1–PM	1.4 ^bc^	14.0 ^a–d^	37.2 ^b–e^	68.3 ^c^	35.6 ^c–g^	133.2 ^e–g^	45.9 ^de^	24.9 ^b–f^	96.7 ^b–d^
	F-LSD _(0.05)_	0.5	2.2	4.3	22.1	19.8	91.6	15.0	4.2	21.4

Means with the same letter(s) within the same column are not significantly different at *p* ≤ 0.05, NHS = non-heat stressed environment, HS = heat stressed environment, WE3 = WE3128, WE5 = WE5323, ZM1 = ZM1523, MF = Mineral fertilizer, MPM = mineral fertilizer + poultry manure, and PM = poultry manure, NoC = cob number, CL = cob length (cm), CW = cob width (mm), CWt = cob weight (g plant^−1^), GWt = grain weight (g plant^−1^), NoG = grain number, SP = shelling percentage (%), SWt = 100-seed weight (g plant^−1^), and SDWt = stover dry weight (g plant^−1^).

## Data Availability

The data presented in this study are available on request from the corresponding author.

## Appendix A

**Table A1:** Physical and chemical properties of the two soil samples.

	Molelwane Soil (Hutton Form)	Tlapeng Soil (Arcadia Form)
Particle size distribution		
Sand %	82	64
Silt %	4	8
Clay %	14	28
Textural class	Loamy sandy	Sandy clay loam
Chemical properties		
pH (KCl) 1:2.5	4.98	5.83
P (Bray1) (mg kg^−1^)	11	2
K (mg kg^−1^)	290	138
Ca (mg kg^−1^)	390	2000
Mg (mg kg^−1^)	163	1310
Na (mg kg^−1^)	5	215
Total N (mg kg^−1^)	376	616
%C	0.37	0.76
S-value	4	22
Ca %	48	45.2
Mg %	33.2	49
K %	18.3	1.6
Na %	0.5	4.2
Ext. acidity (cmol kg^−1^)	0.04	0.036
